# Rescue of common and rare exon 2 skipping variants of the *GAA* gene using modified U1 snRNA

**DOI:** 10.1186/s10020-025-01090-z

**Published:** 2025-02-04

**Authors:** Paolo Peruzzo, Natascha Bergamin, Martina Bon, Sara Cappelli, Alessandra Longo, Elisa Goina, Cristiana Stuani, Emanuele Buratti, Andrea Dardis

**Affiliations:** 1 Regional Coordinator Centre for Rare Diseases, University Hospital of Udine, P. Le Santa Maria Della Misericordi 15, 33100 Udine, Italy; 2 International Centre for Genetic Engineering and Biotechnology (ICGEB), Area Science Park, Trieste, Italy

**Keywords:** Pompe disease, Glycogenosis type II, GAA, Splicing, U1, c.-32-13T>G

## Abstract

**Background:**

Pompe disease (PD) is an autosomal recessive lysosomal storage disorder caused by the deficient activity of acid alpha glucosidase (GAA) enzyme due to mutations in the *GAA* gene. As a result, undigested glycogen accumulates within lysosomes causing their dysfunction. From a clinical point of view, the disease can be classified in infantile-onset (IO) and late-onset (LO) forms. The common *GAA* c.-32-13T>G variant, found in 40–70% of LO-PD alleles, is a leaky splicing mutation interfering with the correct *GAA* exon 2 recognition by the spliceosome leading to the production of non-functional *GAA* transcripts. In this study, we used modified, *GAA*-tailored U1 snRNAs to correct the aberrant splicing determined by the c.-32-13T>G and other *GAA* exon 2-skipping mutations.

**Methods:**

A set of constructs expressing 5 different engineered U1 snRNAs was generated. A functional splicing assay using a *GAA* hybrid minigene carrying different variants known to affect *GAA* exon 2 splicing was used to test the effect of engineered U1 snRNAs on exon 2 inclusion. The effect on endogenously expressed *GAA* transcript and GAA enzymatic activity was assessed by transfecting patient-derived fibroblasts bearing the common c.-32-13T>G with the best performing modified U1 snRNA.

**Results:**

Modified U1-3, U1+1 and U1+6 snRNAs were all able to increase, in a dose-dependent manner, the inclusion of exon 2 within the transcript derived from the *GAA* minigene harbouring the c.-32-13T>G variant. The U1+1 was the most effective one (2,5 fold increase). Moreover, U1+1 snRNA partially rescued the correct splicing of *GAA* minigenes harbouring mutations that affect the 3’ss (c.-32-3C>G, c.-32-2A>G) and the 5’ss (c.546G>A, c.546G>C, c.546G>T). Notably, the treatment of patient-derived fibroblasts carrying the c.-32-13T>G mutation with the U1+1 snRNA increased the amount of normal *GAA* mRNA by 1,8 fold and the GAA enzymatic activity by 70%.

**Conclusions:**

we provide the proof-of-concept for the use of modified *GAA*-tailored U1 snRNAs, designed to potentiate the recognition of the *GAA* exon 2 5’ss, as therapeutic tools to correct the aberrant transcripts carrying variants that affect exon 2 splicing, including the common c.-32-13T>G variant.

**Supplementary Information:**

The online version contains supplementary material available at 10.1186/s10020-025-01090-z.

## Background

Pompe disease – or glycogenosis type II – (PD, MIM 232300) is an autosomal recessive lysosomal storage disorder (LSD), caused by the deficient activity of the acid alpha glucosidase (GAA) enzyme due to pathogenic variants in the *GAA* gene (MIM 606800; NM_000152.5). The enzyme is deputed to the degradation of lysosomal glycogen. Therefore, its deficiency results in the progressive accumulation of unprocessed glycogen within the lysosomes, leading to lysosomes engulfment and dysfunction, especially in cardiac and skeletal muscles (Kohler et al. 2018).

From a clinical point of view, PD patients display extremely variable phenotypes: babies suffering from the severe classic infantile-onset (IO) form typically die for cardio-respiratory complications within the first year of life if not promptly diagnosed and treated, whereas patients affected by the milder, slowly progressive, late-onset (LO) form present features of limb-girdle myopathy and respiratory distress eventually becoming wheel chair- and ventilator-dependent. These latter patients retain some residual GAA enzymatic activity (up to 30% of the activity found in healthy controls), mainly because they bear a *GAA* variant with a milder effect on the enzyme function in at least one allele. However, the clinical presentation only partially correlates with the genotypic asset, suggesting that epigenetic and environmental factors, as well as other genetic modifiers, may also influence the phenotype (Wens et al. 2013; Filippi et al. 2014; Bergsma, et al. 2019).

Enzyme replacement therapy (ERT) using recombinant human GAA protein is the only approved treatment for PD and surely represents a life-saving option for IO-PD patients. However, this therapy is not as effective in LO-PD patients. Indeed, the skeletal muscle seems to be more refractory to therapy due, at least in part, to the autophagic build up observed in muscle fibers which has a profound effect on recombinant enzyme trafficking and processing (Lim et al. 2018).

The *GAA* gene maps to the long arm of the chromosome 17 (17q25.2–25.3) and is composed of 20 exons, the first of which is non-coding, while the initial ATG codon is located 33 bp downstream of the first nucleotide of exon 2. The gene is transcribed in a 3751 bp long mature mRNA encoding for the 952 aa full-length GAA precursor that undergoes extensive processing (N-glycosylation, M6P addition, and proteolytic cleavage) to form the fully active 70 kDa lysosomal enzyme (Wisselaar et al. 1993).

To date, 739 *GAA* variants have been classified as “damaging” in Human Gene Mutation Database (HGMD, www.hgmd.cf.ac.uk), most of them being missense/nonsense mutations (412–55,8%), splicing variants (86–11,6%), small deletions (135–18,3%) and small insertions (55–7,4%). Altogether, small indels, gross deletions and insertions, complex rearrangements and variations in regulatory regions account for the remaining 6,9%. Most of these pathogenic variants, spanning over the entire gene sequence, are private or encountered in a restricted number of families. However, the c.-32-13T>G transversion represents a unique exception, since it is observed in 40–70% of the alleles in LO-PD patients (Peruzzo et al. 2019). The *GAA* c.-32-13T>G variant is defined as a leaky splicing mutation that interferes with the correct processing of *GAA pre*-mRNA resulting in the production of variable amounts of aberrant splicing isoforms, which are characterized by the complete or partial exclusion of exon 2 from the mature *GAA* mRNA (Huie et al. 1994; Boerkoel et al. 1995).

The splicing of *GAA* exon 2 is a complex process whose efficiency is suboptimal per se, as demonstrated by the fact that small amounts of aberrant *GAA* transcripts lacking – totally or partially – the exon 2 can be detected even in fibroblasts derived from healthy individuals. Conversely, in fibroblasts from patient harboring the c.-32-13T>G variant, a little amount of normally spliced *GAA* mRNA is still produced while transcripts lacking exon 2 are the most abundant isoforms. The major functional consequence of this mutation is, therefore, the shifting of the relative proportion between the normal, exon 2-included, *GAA* mRNA and its aberrant exon 2-excluded forms. These non-functional transcripts derive, in part, from the alternative use of a cryptic donor and a cryptic acceptor splice sites (located in intron 1 and within exon 2, respectively) which are favored by the presence of the c.-32-13T>G variant (Dardis et al. 2014).

The splicing of *pre*-mRNA starts with the correct recognition of 5' (5'ss) and 3' (3'ss) splice sites by basic components of the spliceosome. Specifically, the 5’ss is recognized by the U1 small nuclear ribonucleoprotein (snRNP), whose RNA component (U1 snRNA) is able to base-pair to specific consensus sequences that determine the strength of the 5'ss (Roca et al. 2013). However, the overall splicing efficiency of a given exon might be heavily influenced by the presence of several other factors such as, for example, cryptic splice sites and exonic and intronic silencer and enhancer elements (Conti et al. 2013; Buratti and Baralle 2010). In this complex scenario, it is therefore not surprising that the effect of genetic variants that possibly perturb a splicing-relevant region of a gene is not easy to predict a priori.

In recent years, an innovative therapeutic tool based on modified U1 snRNA has been developed to restore the correct splicing in several genetic pathologies for which exon-skipping defects have been described (Rogalska et al. 2016; Gonçalves et al. 2023). These engineered U1 snRNAs are 165 bp long RNA that differ from the endogenous U1 snRNA for their 5’ end that is designed to bind downstream the 5’ splice site of the defective exon, promoting the targeted recruitment of the spliceosome machinery with a consequent improvement of splicing efficiency (Matos et al. 2022). Here, we have explored the possibility to apply this strategy to correct the aberrant splicing caused by the common c.-32-13T>G variant using an *in-vitro GAA* minigene approach and patient’s fibroblasts. Moreover, we tested a set of *GAA* mutations previously reported to cause exon 2 skipping, identifying several known disease-associated variants that could potentially benefit from the modified U1 snRNA treatment (Goina et al. 2019).

## Materials and methods

### Primary fibroblasts isolation and cell culture conditions

Primary fibroblasts were isolated from the skin biopsy of a PD patient carrying the *GAA* c.-32-13T>G variant in compund heterozygosis with a second, still unidentified null mutation. The patient signed the informed consent for research purpose of his biological material. The fresh biopsy was washed in PBS and chopped into small pieces under sterile conditions. Using a sterile glass coverslip, four to six pieces per well were forced to adhere to the surface of a 6-well plate and left untouched in complete medium [DMEM High Glucose (Euroclone – Milan, Italy) supplemented with 10% Foetal Bovine Serum (Gibco), 2 mM glutamine, 100 UI/mL penicillin and 100 µg/mL streptomycin (Euroclone – Milan, Italy)] for approximately 2 weeks, until the first fibroblasts begun to spread from the biopsy masses. The culture medium was then replaced every 3 days until the cell density reached the subculturing conditions. Primary fibroblasts were used before their tenth passage for the experiments. Both primary and human telomerase (h-Tert)-competent, immortalized fibroblasts, as well as HeLa and Hek 293 cell lines, were routinely cultured in complete medium and maintained at 37 °C in a humidified atmosphere enriched with 5% (v/v) CO_2_.

### GAA mutant minigenes and GAA-tailored U1 snRNA plasmids

The *GAA* WT minigene plasmid, from which all the *GAA* mutant minigenes were derived by site-directed mutagenesis (Goina et al. 2019), has already been extensively described in our previous publications (see reference 10 for a detailed description). Starting from the parental pG3-U1wt vector (Pagani et al. 2002), the *GAA*-tailored U1-7, U1-3, U1+1 and U1+6 snRNA plasmids were obtained by replacing the sequence between *BclI* and *BglII* restriction sites with specific mutant oligonucleotides.

### Minigene/U1 plasmid transient co-transfection

6 × 10^5^ Hek 293 or 3,5 × 10^5^ HeLa cells/well were seeded in 6-well plates 24 h before transfection. 0,5 µg of MUT (or WT) minigene were co-transfected with 1,5 µg (for single-dose experiments) or progressive amount (0,5 – 1 – 2 µg, for dose-escalation experiments) of the specific U1 vector using Lipofectamine 2000 (Invitrogen – Waltham, Massachusetts) following manufacturer’s protocol. 24 h after transfection, total RNA was extracted using RNeasy mini kit (Qiagen – Hilden, Germany) and, for each sample, an on-column DNase digestion was performed. 1 µg of total RNA was reverse transcribed using SuperScript IV First-strand synthesis kit (Invitrogen – Waltham, Massachusetts) and the Glo800Rbis minigene-specific primer. The obtained cDNA was used for the minigene functional splicing assay or real-time qPCR experiments.

### *U1* + *1 snRNA plasmid transient transfection in immortalized c.-32-13T*>*G fibroblasts*

6,5 × 10^5^ patient-derived immortalized fibroblasts carrying the *GAA* c.-32-13T>G variant were seeded in a 10 cm-diameter Petri dish 24 h before transfection. 5,8 µg (low dose) or 11,6 µg (high dose) of U1+1 modified snRNA plasmid were transiently transfected using Lipofectamine 2000 (Invitrogen – Waltham, Massachusetts) following manufacturer’s instructions. Immortalized fibroblasts transfected with 5,8 µg of U1 wt plasmid were considered as the negative snRNA control condition while mock-transfected fibroblasts were used as reference sample. 48 h after transfection, total RNA was extracted using RNeasy mini kit (Qiagen – Hilden, Germany) and, for each sample, an on-column DNase digestion was performed. 1 µg of total RNA was reverse transcribed using random primers and SuperScript IV First-strand synthesis kit (Invitrogen – Waltham, Massachusetts). The obtained cDNA was used for subsequent end-point PCR or real-time qPCR experiments.

### *U1* + *1 snRNA plasmid electroporation in primary c.-32-13T*>*G fibroblasts*

U1+1 snRNA plasmid electroporation in primary c.-32-13T>G fibroblasts was performed using P2 Primary Cell 4D-Nucleofector™ X Kit (Lonza – Basel, Switzerland) following manufacturer instructions. Briefly, 6,5 × 10^5^ primary fibroblasts were pelletted, resuspended in 100 µL of P2 Primary Cell Nucleofector™ solution containing 5,8 µg (low dose) or 11,6 µg (high dose) of U1+1 modified snRNA plasmid and electroporated in a Nucleocuvette^™^ Vessel using the impulse code EN150 of the 4D-Nucleofector instrument (Lonza – Basel, Switzerland). After 10 min of recovery, the cells were resuspended in 500 µL of pre-warmed complete medium and plated in 10 cm-diameter Petri dish. Primary c.-32-13T>G fibroblasts transfected with 5, 8 µg of U1 wt plasmid were considered as the negative snRNA control condition while mock-electroporated fibroblasts were used as reference sample.

### Minigene functional splicing assay

The simultaneous amplification of the minigene-derived splicing products was obtained using pTB-specific primers Alfa 2–3 FOR and Bra 2 REV and the high-fidelity Platinum™ Taq DNA polymerase (Invitrogen – Waltham, Massachusetts). The following conditions were adopted for the amplification: initial denaturation = 95 °C for 3 min; thermocycling = (95 °C for 30 s, 54 °C for 30 s, 68 °C for 1 min) for 35 cycles; final elongation = 68 °C for 7 min. The PCR products were resolved in a 2% agarose gel to improve the separation of SV2 and SV3 bands.

### Endogenous GAA functional splicing assay

The simultaneous amplification of the *GAA* normal, exon 2-included, splicing isoform (N) and aberrant splicing isoforms with complete (SV2; r.-32_546del) or partial (SV3; r.-32_486del) exclusion of exon 2, was obtained from c.-32-13T>G fibroblast cDNA using GAA WT FOR (exon 1) and GAA SKIP2 REV (exon 3) primers and the GoTaq DNA polymerase (Promega – Madison, Wisconsin). The following conditions were adopted for the amplification: initial denaturation = 95 °C for 3 min; thermocycling = (95 °C for 30 s, 63 °C for 30 s, 72 °C for 1 min) for 40 cycles; final elongation = 72 °C for 7 min. The PCR products were resolved in a 2% agarose gel to improve the separation of SV2 and SV3 bands.

### Real-time qPCR

The magnitude of *GAA* exon 2 inclusion within the splicing products in the minigene-transfected Hek 293 cells over-expressing the different modified U1 snRNAs was quantified by real-time qPCR using the primers GAA mini FOR and GAA mini REV specific for fibronectin EDB and *GAA* exon 2 of the pTB minigene, respectively. The amplification of the minigene-specific Alfa Globin, obtained using the primers Alfa Glo FOR and Alfa Glo REV, was used as internal reference for transfection efficiency. The magnitude of *GAA* exon 2 inclusion in c.-32-13T>G fibroblasts following modified U1+1 snRNAs transfection was achieved by amplifying the *GAA* N isoform using GAA WT FOR (exon 1) and GAA WT REV (exon 2) primers and normalizing for the amount of *HPRT* house-keeping gene, amplified using HPRT FOR and HPRT REV primers. Real-time qPCR were performed using Sso Advance Universal SYBR Green Supermix (Bio-Rad – Hercules, California) and run on the QuantStudio 3 instrument (Applied Biosystems – Waltham, Massachusetts). Data were calculated by applying the ΔΔct equation and considering the minigene alone-transfected Hek 293 cells or the mock-transfected fibroblasts as the reference samples.

### GAA enzymatic activity assay

GAA enzymatic activity was measured using 4-methylumbelliferyl-α-D-glucopiranoside (Glycosynth – Warrington, England) as a fluorogenic substrate. Briefly, 72 h after U1+1 or U1 wt snRNAs transfection/electroporation, c.-32-13T>G fibroblasts were harvested in PBS and the cell pellets were resuspended in 40 μL of water, sonicated at ice-cold temperature and centrifuged. Total protein concentration in cell lysates was measured by Bio-Rad Protein Assay (Bio-Rad – Hercules, California). Then, 20 μg of total protein for each sample were mixed with 10 μL of 2 mM 4-methylumbelliferyl- α -D-glucopiranoside in acetate buffer in a final volume of 20 μL and incubated at 37 °C for 2 h. The reaction was stopped by the addition of 1,98 mL of 0,5 M carbonate buffer (pH 10.7). The resulting fluorescence was recorded at 355 and 460 nm excitation (Ex) and emission (Em) using a Gemini microplate reader (Molecular Devices – San Jose, California). All assays were performed in triplicate.

### Statistical analysis

Shapiro–Wilk’s test was performed for each experimental dataset to check for the normal distribution of the data using GraphPad Prism software (version 8.0.2). As all the datasets resulted to be normally distributed, the unpaired Student t-test was employed to determine the level of statistical significance within the experiments.

### Oligonucleotides

The oligonucleotides used in this work, all purchased from Sigma-Aldrich (Darmstadt, Germany), are summarized in the following table:

**Table:** 

	Primer	Sequence (5'—3')
Reverse transcription (minigene-specific)	Glo800Rbis	CACAGAAGCCAGGAACTTGTCC
Minigene functional splicing assay in HeLa and Hek 293 cell lines	Alfa 2–3 FOR	CAACTTCAAGCTCCTAAGCCACTGC
	Bra 2 REV	TAGGATCCGGTCACCAGGAAGTTGGTTAAATCA
Endogenous *GAA* splicing assay in primary fibroblasts	GAA WT FOR	CCACCTCTAGGTTCTCCTCGT
	GAA SKIP2 REV	CGGAGAACTCCACGCTGTA
Real-time qPCR (minigene-specific)	GAA mini FOR	CCCGGCCTGGAGTACAATG
	GAA mini REV	CAGGAGTGCAGCGGTTGC
	Alfa Glo FOR	ACCAAGACCTACTTCCCGCACTTCG
	Alfa Glo REV	CAGGCAGTGGCTTAGGAGCTTGAAG
Real-time qPCR (endogenous *GAA*)	GAA WT FOR	CCACCTCTAGGTTCTCCTCGT
	GAA WT REV	TCCTACAGGCCCGCTCC
	HPRT FOR	GACCAGTCAACAGGGGACAT
	HPRT REV	GTGTCAATTATATCTTCCACAATCAAG

## Results

### Effect of modified U1 snRNAs on exon 2 inclusion in a minigene bearing the c.-32-13T>G *GAA* mutation

**Fig. 1:**
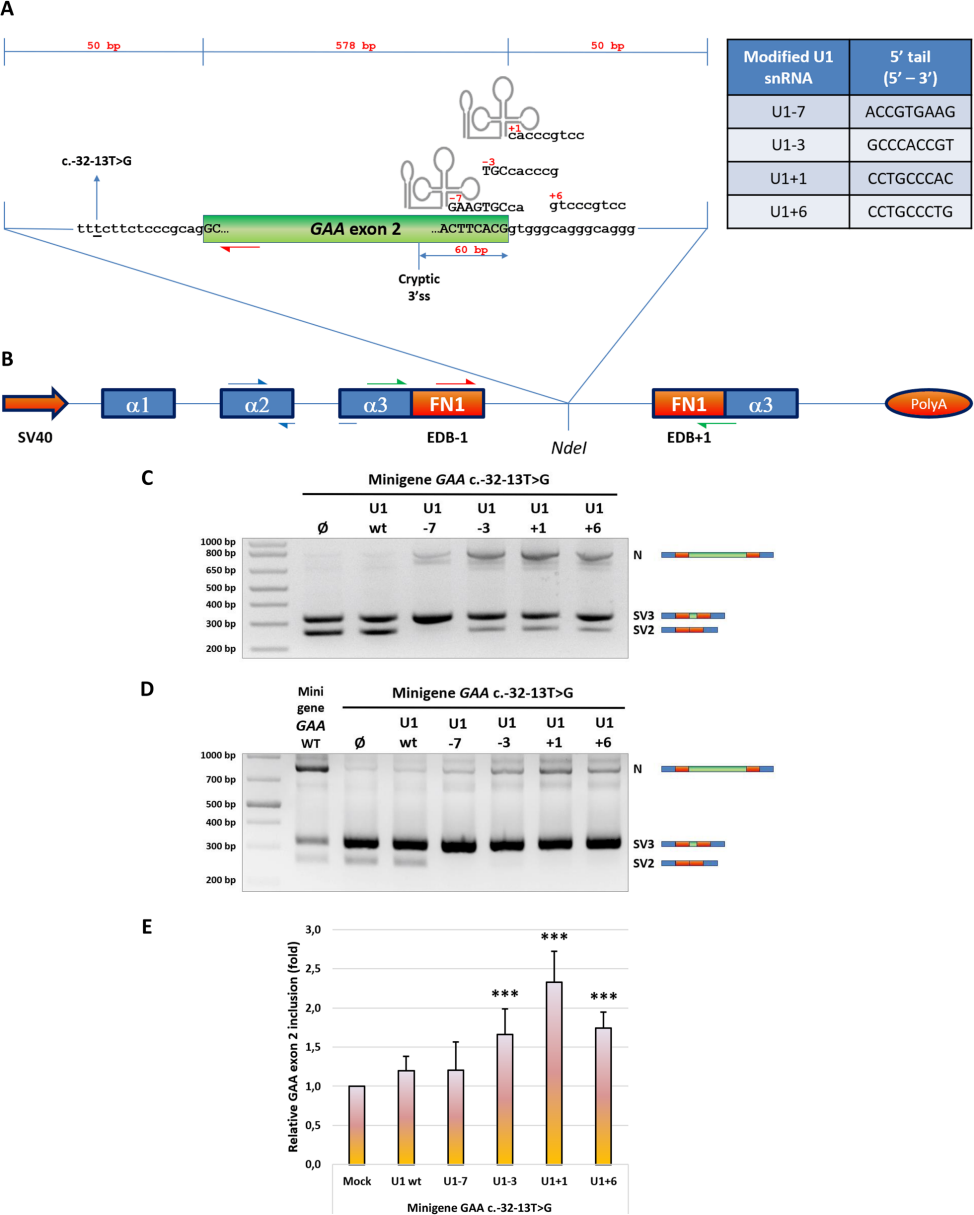
Effect of modified U1 snRNAs −7, −3, +1 and +6 on the splicing of *GAA* c.-32-13T>G (MUT) minigene. **A** The schematic diagram shows the *GAA* minigene sequences targeted by the modified U1 snRNAs. The table reports, for each modified U1 snRNA, also the specific 9 bp-long tail sequence (5’ to 3’) that ensures complementarity to the selected positions within *GAA* intron 2 donor splice site. **B** Schematic representation of the human *GAA* minigene system: the entire *GAA* exon 2 (green box) and 50 nucleotides of both flanking introns were cloned within the *NdeI* restriction site in the splicing-proficient pTB vector containing the Alfa Globin (blue boxes) and fibronectin extra-domain B (EDB - red boxes) exons. The MUT minigene harbouring the *GAA* c.-32-13T>G variant (underscored) was obtained by site-directed mutagenesis from the *GAA* WT minigene. The position of the cryptic 3’ splice site 60 bp upstream the end of *GAA* exon 2 is highlighted. Primers Alfa 2–3 FOR and Bra 2 REV used for the minigene functional splicing assay are represented by green half-arrows, whereas primer couples GAA mini FOR/GAA mini REV and Alfa Glo FOR/Alfa Glo REV used for the quantification of *GAA* exon 2 inclusion by real-time qPCR are depicted by red and blue half-arrows, respectively. **C** and **D** Functional splicing assay of MUT minigene after modified U1 snRNAs co-transfection performed on HeLa and Hek 293 cell lines, respectively. The RT-PCR amplified bands correspond to the normal, *GAA* exon 2-included, splicing isoform (N) and the aberrant splicing isoforms with complete (SV2; r.-32_546del) or partial (SV3; r.-32_486del) exclusion of *GAA* exon 2, the latter deriving from the activation of the 3’ cryptic splice site. *GAA* WT minigene was used as internal control. Agarose gel pictures show a representative result of five independent experiments (**E**) Real-time qPCR analysis of the extent of *GAA* exon 2 inclusion (N isoform) in MUT minigene/modified U1 snRNAs co-transfected Hek 293 cells. Data, expressed as fold of inclusion relative to the MUT minigene alone, are presented as mean ± SD of four independent experiments. Statistical analysis was conducted using Student t-test. *** = p-value < 0,001

As a first step, we evaluated an experimental strategy using exon-specific U1 snRNAs aimed at rescuing the correct 5’ss recognition of the canonical *GAA* exon 2. To maximize the efficiency of U1 snRNP targeting to the canonical 5’ss of *GAA* exon 2, we generated a set of modified U1 snRNAs (namely U1-7, U1-3, U1+1 and U1+6) by engineering their 5’ tail to specifically bind four different sequences within the exon2/intron2 junction, as schematically reported in Fig. 1A. The sequence of these modified U1 snRNAs was cloned into pGEM expression plasmids for transfection in cells.

To assess the efficacy of the modified U1 snRNAs in restoring correct exon 2 inclusion within *GAA* transcript in the presence of the c.-32-13T>G splicing variant, we then adopted an *in-cellulo* approach by exploiting a minigene system already validated and employed in our previous works aimed at characterizing in depth this splicing event (Dardis et al. 2014; Goina et al. 2017). Briefly, the wild-type (WT) *GAA* minigene and its c.-32-13T>G mutated (MUT) counterpart were obtained by cloning the *GAA* exon 2 and 50 nucleotides of both flanking regions within the *NdeI* restriction site in a pTB-based, splicing-competent vector (Fig. 1B). HeLa cells were then co-transfected with 0,5 µg of the MUT minigene and 1,5 µg of the different modified U1s. A functional splicing assay was also performed using cells transfected with the WT minigene as control. As shown in Fig. 1C, in normal conditions the splicing profile of the mutated minigene is characterized by two low-weight splice forms corresponding to the complete (SV2) or partial (SV3) skipping of the exon 2 of *GAA*, the SV3 resulting from the usage of the cryptic 3’ss located 60 bp upstream the end of *GAA* exon 2. As expected, the over-expression of the wt U1 snRNA did not have any effect on *GAA* exon 2 inclusion because it is well known that in eukaryotic cells this factor is massively expressed with a relative abundance of 10^6^ molecules per cell (Baserga et al. 1993). However, the over-expression of the modified U1s was able to partially restored *GAA* exon 2 inclusion. Nevertheless, not all the U1 5’ modifications performed equally well in this context, U1+1 being the most effective one whilst U1-7 was capable of increasing only weakly the rate of *GAA* exon 2 inclusion.

These data were also verified in Hek 293 cell line (Fig. 1D) showing that there did not seem to be a cell-type specific effect. These qualitative data were then confirmed by real-time qPCR experiments specifically designed to detect only the *GAA* exon 2-included minigene transcript. Indeed, an increase of about 2,5 fold in exon 2 inclusion was detected in U1+1 treated cells compared to mock treated ones. Similarly to U1+1, both U1+6 and U1-3 promoted exon 2 inclusion but in a slightly less efficient manner, as demonstrated by an almost 2-fold increase in correctly spliced minigene mRNA. In contrast, no significant splicing improvement was observed when the mutant minigene was co-expressed with wt U1 or modified U1-7 snRNAs (Fig. 1E). Of note, the accuracy of the mutant minigene splicing process upon U1+1 snRNA over-expression was verified by Sanger sequencing the 3’ *GAA*/fibronectin junction within the normal, *GAA* exon 2-included transcript. As demonstrated by the electropherogram reported in the Additional Fig. S1, the inclusion of *GAA* exon 2 within the normal transcript was precisely achieved, without any extra nucleotide belonging to the flanking intron.

**Fig. 2:**
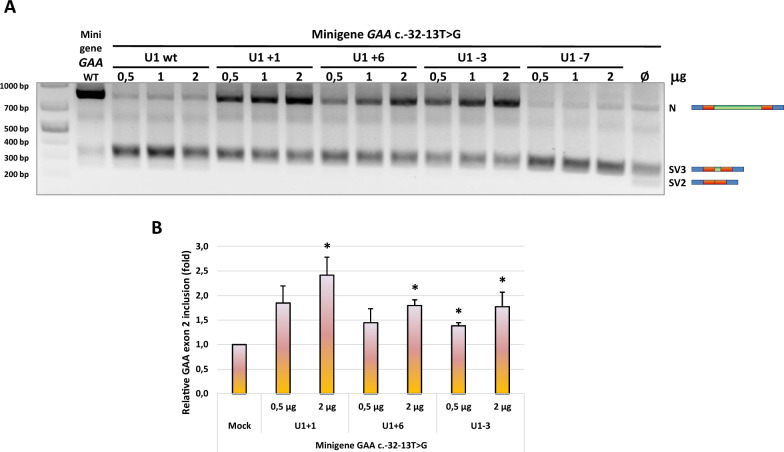
Effect of dose-escalation of modified U1 snRNAs −7, −3, +1 and +6 on the splicing of *GAA* c.-32-13T>G (MUT) minigene. **A** Functional splicing assay performed on Hek 293 cells co-transfected with 0,5 µg of MUT minigene and increasing doses (0,5 – 1 – 2 µg) of the different modified U1 snRNAs. The minigene-specific N, SV3 and SV2 splicing isoforms are evidenced on the right. The agarose gel picture shows a representative result of two independent experiments. **B** Real-time qPCR analysis of the extent of *GAA* exon 2 inclusion (N isoform) in MUT minigene/effective modified U1 snRNAs co-transfected Hek 293 cells. Data, expressed as fold of inclusion relative to the MUT minigene alone, are presented as mean ± SD of two independent experiments. Statistical analysis was conducted using Student t-test. * = p-value < 0,05

To rule out the possibility that the differences in exon inclusion proficiency displayed by the modified U1s might be due to sub-optimal U1 concentrations, we then performed a dose-response experiment by transfecting scalar amount (0,5, 1 and 2 µg) of U1 plasmids in mutant minigene-expressing Hek 293 cells (Fig. 2). Once again, the splicing profile of c.-32-13T>G minigene in cells treated with increasing doses of modified U1s confirmed the effectiveness of U1+1, U1+6 and U1-3 in promoting the inclusion of *GAA* exon 2 in the mature minigene mRNA and the lack of promotion by U1-7. Moreover, the rate of exon inclusion qualitatively correlated with U1 concentration in a dose-dependent fashion (Fig. 2A). Accordingly, real-time qPCR performed on minigene-transfected cells overexpressing the effective modified U1s showed that the highest rate of *GAA* exon 2 inclusion was achieved when 2 µg of each U1 plasmid were transfected (Fig. 2B). No statistically significative differences in *GAA* exon 2 inclusion were observed between using 1,5 and 2 µg of U1 plasmids in the co-transfections, demonstrating that the diverse efficacy of the different modified U1s was not due to quantity issues. Altogether, these results suggest that targeting early spliceosome assembly machinery to specific sequences within *GAA* exon2/intron2 junction could represent a valuable therapeutic approach to improve the splicing efficiency of *GAA* mRNA in the presence of c.-32-13T>G variant.

### Effect of modified U1+1 snRNA on exon 2 inclusion in minigenes bearing different exon 2 *GAA* splicing variants

**Fig. 3:**
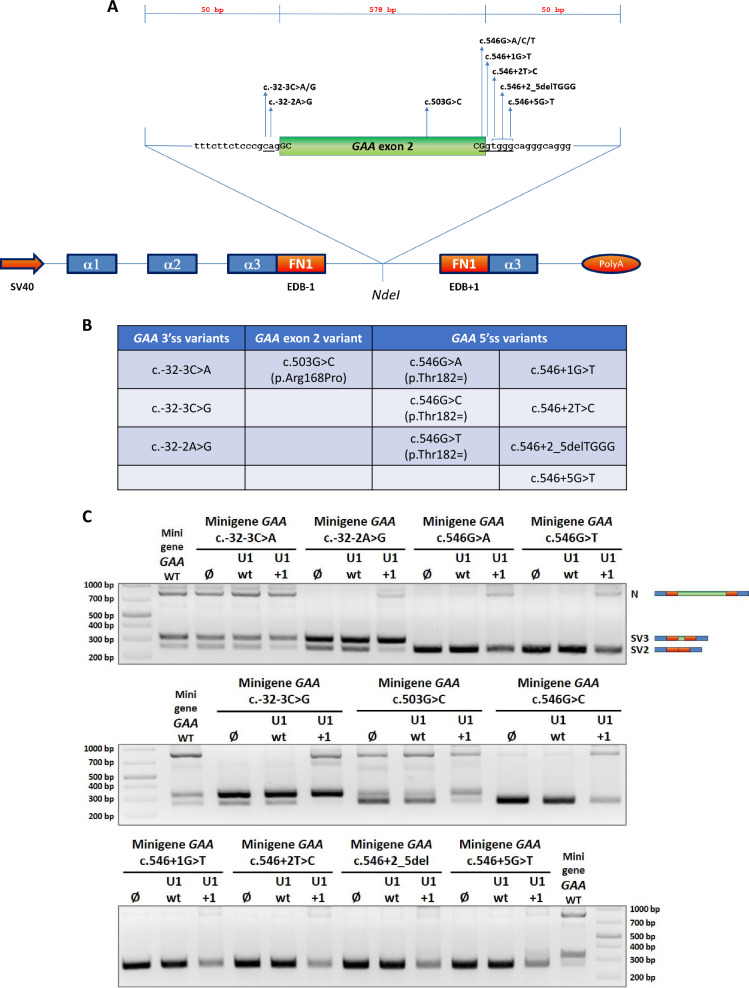
Effect of modified U1+1 snRNA on the splicing of mutant *GAA* minigenes harbouring the 11 selected *GAA* splicing-affecting variants. **A** The cartoon indicates the position of the 11 selected *GAA* splicing variants within the *GAA* insert of the minigene plasmid. All the *GAA* mutant minigenes were obtained by site-directed mutagenesis from the *GAA* WT minigene (Goina E et al*.*, 2019). (**B**) The table reports the distribution of the 11 selected *GAA* splicing variants: three of them (c.-32-3C>A, c.-32-3C>G and c.-32-2A>G) fall within the 3’ splice site; one (c.503G>C) is exonic and seven (c.546G>A, c.546G>C, c.546G>T, c.546+1G>T, c.546+2T>C, c.546+2_5delTGGG and c.546+5G>T) involve the 5’ splice site. **C** Functional splicing assay performed on Hek 293 cells co-transfected with 0,5 µg of the reported *GAA* mutant minigene and 1,5 µg of wild-type U1 or modified U1+1 snRNAs. For each set, the transfection with the minigene alone was considered the reference sample. *GAA* WT minigene was used as internal control in each agarose gel. The minigene-specific N, SV3 and SV2 splicing isoforms are evidenced on the right

Although the most common mutation in LO-PD patients, it is important to note that the c.-32-13T>G is not the only splicing-affecting variant leading to an impairment of the *GAA* exon 2 inclusion in the mature transcript (Pompe database – www.pompevariantdatabase.nl). We have recently documented the aberrant splicing pattern of several putative splicing variants within *GAA* exon 2 and flanking introns and demonstrated their responsiveness to an Antisense Morpholino Oligonucletotides (AMOs)-based treatment (Goina et al. 2019). Therefore, we sought to address the possibility of applying the modified U1-based strategy to correct the aberrant splicing caused by some of these mutations. Considering the severity of the splicing defect, we therefore selected 11 splicing variants that could potentially benefit from this experimental approach: three of them fall in the 3’ acceptor splice site of exon 2, one is located within the exon 2, and seven affect the 5’ donor splice site of exon 2 (Fig. 3A and B). With an experimental setting like the one adopted for testing the modified U1s efficacy in the context of the c.-32-13T>G variant, we performed the functional splicing assay by co-transfecting Hek 293 cells with a set of minigenes harboring each of the 11 selected *GAA* splicing variants and the pGEM vector coding for U1+1 snRNA (the most effective one) or U1 wt as control. As shown in Fig. 3C, each mutated minigene displayed a specific splicing profile that was never modified, in all cases, by the over-expression of the wild-type form of U1 snRNA. On the contrary, U1+1 snRNA generally exerted a positive effect in increasing the inclusion rate of *GAA* exon 2 in the mature transcript with some exceptions. For example, U1+1 snRNA treatment nicely rescued the correct splicing of the one bearing the c.-32-3C>G. Interestingly, a different nucleotide change at the same position (c.-32-3C>A) resulted in a completely different minigene splicing pattern was not substantially altered by U1+1 snRNA treatment. Moreover, we also observed a slighter increase in the amount of correctly-spliced transcript was observed in c.-32-2A>G mutant minigene following U1+1 snRNA treatment, showing that there is a limit to the use of modified U1 snRNAs depending on how serious is the effect of the nucleotide change on 3’ss definition.

Another interesting result from this analysis occurred for the case of the c.503G>C exonic variant, where the main effect of U1+1 was the promotion of the cryptic 3’ss usage, as shown by the very prominent production of the SV3 isoform and with no appreciable increase in the normally spliced isoform. This result highlights the well-known combinatorial regulation property of the splicing process, where local sequence context can also play a role in deciding the final outcome (Spena et al. 2006).

On the contrary, every nucleotide variation affecting the 5’ donor splice site of exon 2 resulted in the SV2 isoform being the only species prominently affected. In fact, in all cases when these minigenes were co-transfected with U1+1 a reduced amount of SV2 isoform was observed. Nonetheless, U1+1 treatment resulted in a partial rescue of exon 2 inclusion in transcripts carrying variants affecting the last nucleotide of exon 2 (c.546G>A, c.546G>C and c.546G>T), whilst very little effect was observed in transcripts carrying variants that generate mismatch(es) with respect to the U1+1 modified 5’ end.

### Effect of modified U1+1 snRNA on exon 2 inclusion in patient-derived fibroblasts carrying the *GAA* c.-32-13T>G splicing variant.

**Fig. 4:**
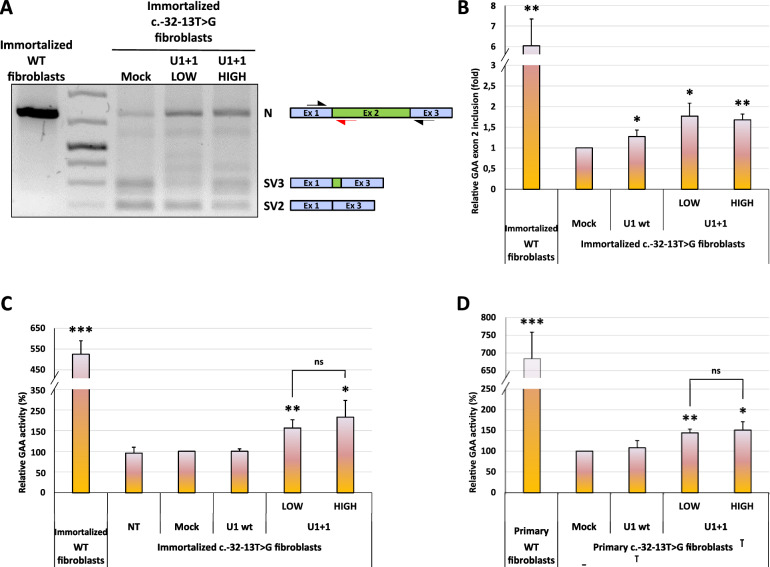
Effect of modified U1+1 snRNA on the splicing of endogenous *GAA* in immortalized and primary c.-32-13T>G fibroblasts. **A **Functional splicing assay performed on endogenous *GAA* mRNA in patient-derived immortalized fibroblasts carrying the c.-32-13T>G variant transfected with 2 doses (LOW = 5,8 g; HIGH = 11,6 g) of modified U1+1 snRNA plasmid. The mock-transfected fibroblasts were considered as the reference sample. The *GAA* N, SV3 and SV2 splicing isoforms and the position of the primers GAA WT FOR and GAA SKIP2 REV (black half-arrows) are evidenced on the right. The red half-arrow represents the GAA WT REV primer used, in combination with the GAA WT FOR primer, to quantify the endogenous *GAA* N isoform in real-time qPCR experiments. **(B)** Real-time qPCR analysis of the extent of *GAA* exon 2 inclusion (N isoform) in c.-32-13T>G fibroblasts transfected with modified U1+1 snRNAs at low and high doses. The transfection with U1 wt was used as negative snRNA control condition. The data, normalized to the expression of *HPRT* house-keeping gene and displayed as fold of inclusion relative to the mock-transfected c.-32-13T>G fibroblasts, are presented as mean ± SD of three independent experiments. Statistical analysis was conducted using Student t-test. * = p-value < 0,05; ** = p-value < 0,01. **C** GAA enzymatic activity measured in immortalized c.-32-13T>G fibroblasts transfected with U1+1 snRNA at low and high doses. The transfection with U1 wt was used as negative snRNA control condition. Data, expressed as percentage increase relative to the mock-transfected c.-32-13T>G fibroblasts, are presented as mean ± SD of three independent experiments. Statistical analysis was conducted using Student t-test. * = p-value < 0,05; ** = p-value < 0,01; *** = p-value < 0,001; ns = not significant. **D** GAA enzymatic activity measured in primary c.-32-13T>G fibroblasts electroporated with U1+1 snRNA at low and high doses. The electroporation with U1 wt was used as negative snRNA control condition. Data, expressed as percentage increase relative to the mock c.-32-13T>G fibroblasts, are presented as mean ± SD of three independent experiments. Statistical analysis was conducted using Student t-test. * = p-value < 0,05; ** = p-value < 0,01; *** = p-value < 0,001; ns = not significant. Throughout the figure, the values obtained in wild type immortalized (panel A, B and C) and primary (panel D) fibroblasts were reported for comparison

Considering the promising effects exerted by U1+1 snRNA on *GAA* exon 2 splicing in the minigene system, we decided to assess its efficacy also in immortalized fibroblasts derived from a PD patient carrying the c.-32-13T>G variant in compound heterozygosis with a second null mutation. The utility in using this cellular model to evaluate *GAA* exon 2 splicing profile relies on the fact that every possible effect of U1+1 snRNA eventually observed, can be specifically related to the correction of the c.-32-13T>G splicing defect, without confusing interferences caused by pre-mRNA production from the second allele. Therefore, we transfected these cells with the plasmid coding for U1+1 snRNA at two different doses (low = 5,8 μg and high = 11,6 μg) to explore a potential dose/effect correlation and performed a functional splicing assay using *GAA*-specific primers. Interestingly, as shown in Fig. 4A, the over-expression of U1+1 snRNA at both concentrations resulted in the improvement of *GAA* exon 2 splicing efficiency, as demonstrated by the increased intensity of the N band coupled, in parallel, to a decreased production of SV3 (low dose) and SV2 (high dose) aberrant isoforms compared with mock-transfected immortalized fibroblasts.

These qualitative data were further confirmed by real-time qPCR experiments specifically designed to quantify the amount of the normally spliced *GAA* transcript that resulted, independently of the employed dose, in about 1,8 fold increase in U1+1-treated immortalized fibroblasts compared to that measured in mock-transfected c.-32-13T>G cells (Fig. 4B). To test whether this U1+1-dependent improvement in the *GAA* exon 2 splicing translated in a downstream functional impact, we measured the GAA enzymatic activity in U1+1-transfected cells. As shown in Fig. 4C, transfected cells displayed a 70% increased activity compared to mock- or U1 wt-transfected cells (Fig. 4C). Moreover, the transfection condition with a low dose of U1+1 snRNA was already enough to reach this increment as the treatment with the high dose of U1+1 snRNA did not further increase the GAA enzymatic activity. This result was further confirmed in c.-32-13T>G primary fibroblasts that undergone electroporation to allow the expression of U1+1 modified snRNA (Fig. 4D). The *GAA* genotype and expression levels of these primary cells have been extensively characterized in our previous work (Dardis et al. 2014). Similarly to what it was observed in immortalized patient fibroblasts, the GAA enzymatic activity resulted 50% higher in U1+1-expressing primary fibroblasts – even at the lowest U1+1 dose – compared to mock- or U1 wt-expressing counterparts.

Finally, in order to explore the specificity U1+1 snRNA, we adopted a multistep bioinformatic approach aimed at the identification of the potential U1+1 off-target transcripts (Additional Fig. 2A). In particular, we used BLASTN (https://blast.ncbi.nlm.nih.gov/Blast.cgi) to recover all the human transcripts from the NCBI RefSeq_Select database that match 100% with the 9 bp-long U1+1 target sequence (GTGGGCAGG) present at the 5’ splice site of *GAA* exon 2. This interrogation returned 1787 matches, 736 of which presented the target sequence within their coding region. We then ranked these 736 genes on the basis of how many times the target sequence was present within each transcript and selected the genes bearing the U1+1 target sequence 3 times within the coding region (*CBX2*, *OBSCN* and *CSPG4*) as the most likely U1+1 off-target genes. Therefore, we decided to test the expression of these 3 genes in patient-derived primary fibroblasts after the U1+1 snRNA electroporation, using U1 wt as the negative snRNA control (Additional Fig. 2B). While no change in *OBSCN* expression levels was evident in U1+1-expressing fibroblasts compared to mock control, we noticed a 40% decrease in *CBX2* and *CSPG4* expression levels. However, as the variations observed following U1+1 snRNA treatment were very similar to those observed using the U1 wt negative snRNA control, we concluded that these expression changes were not U1+1-specific.

## Discussion

Since the initial approval in 2006 of ERT with alglucosidase alfa, the recombinant form of human *GAA*, the standard of care for the treatment of PD has been the intravenous administration of this recombinant enzyme twice a month. This therapy has proved to be effective in improving cardiac and respiratory functions, as well as motor skills, in babies affected by the severe IO form of the disease and ultimately prolonging their survival especially when the treatment is started before six months of age (Kishnani et al. 2007; Yang et al. 2016). In contrast, the clinical outcomes of LO-PD patients on ERT, albeit generally positive, are more variable in terms of benefit duration and force vital capacity (Schoser et al. 2017; Dornelles et al. 2021; Claeys et al. 2022). In recent years, several efforts in many different research fields have been made to broaden the therapeutic options for the treatment of PD (Bellotti et al. 2020). With the perspective of a personalized medicine, novel genetic-based approaches using antisense oligonuleotides or splicing-modulating small molecules have been proposed to restore normal splicing of *GAA* transcripts carrying different splicing mutations, including the common c.-32-13T>G variant (Goina et al. 2017; Wal et al. 2017; Buratti et al. 2020).

As already mentioned above, even in the absence of mutations, the splicing efficiency of exon 2 of *GAA* is not optimal. Indeed, both 3’ acceptor splice site and the 5’ donor splice site of this exon are predicted to be poorly defined. In particular, the degenerated *GAA* exon 2 5’ss (ACG|GUGGGC) belongs to the 5% of global 5’ss that differ by 4 nucleotides from the conserved canonical sequence (CAG|GURAGU) defining the exon/intron boundary (Carmel et al. 2004), likely accounting, at least in part, for the suboptimal splicing efficiency of this exon.

As can be expected from this weakness, it is not surprising that out of the 86 pathogenic splicing variants documented to date in the *GAA* gene, 15 affect the splicing process of exon 2 (HGMD, www.hgmd.cf.ac.uk).

Based on these considerations, in this work we tested the feasibility of using a U1 snRNP-based approach for the functional correction of exon 2 aberrant splicing determined by pathogenic variants in PD. This kind of therapeutic strategy, oriented toward a better exon definition through a precise 5’ss recognition, has already been successfully experimented for the correction of splicing-related variants in different pathologies such as spinal muscular atrophy (Mas et al. 2015), familial dysautonomia (Donadon et al. 2018) and coagulation factor VII deficiency (Pinotti et al. 2008), among others.

To achieve our aim, a set of 4 different U1 snRNA molecules was designed to accurately target the 5’ss of *GAA* exon 2 by modifying their 5’ tail in order to match the mRNA region between nucleotides −7 and +6. We focused our attention, at first, on the c.-32-13T>G splice variant, a mutation that, by perturbing the polypyrimidine tract located upstream *GAA* exon 2, abrogates the binding of the U2AF65 splicing factor leading to *GAA* misplicing (Dardis et al. 2014). This variant is the most frequent *GAA* mutation carried by 90% of PD patients manifesting the LO phenotype (Reuser et al. 2019). Our results, obtained through an *in-cellulo* functional splicing assay, evidenced a positive effect of all engineered U1s in rescuing the inclusion of *GAA* exon 2 within the mature mutant minigene transcript (although to different extents). In particular, a modified U1+1 snRNA showed the most promising, dose-response effect: its use resulted in a 2,5 fold increase in *GAA* exon 2 rate of inclusion, while it completely abolished the production of the SV2 non-functional splicing isoform. Like all other modified U1 tested, however, the U1+1 snRNA did not alter the detrimental usage of the *GAA* exonic cryptic 3’ss, maintaining unaltered the generation of the aberrant SV3 splicing isoform. Most importantly, the U1+1 snRNA seemed to be effective also in PD patient-derived immortalized fibroblasts carrying the c.-32-13T>G common variant. In these cells, the partial rescue of the normally spliced *GAA* mRNA was already achieved with the lowest U1+1 snRNA concentration. However, incrementing the amount of U1+1 snRNA did not further increase the inclusion of *GAA* exon 2. This was somehow unexpected, given the good dose-response correlation observed in terms of *GAA* exon 2 inclusion in HEK 293 cells transfected with the mutant hybrid minigene. We believe that the reason lies in the fact that, in HEK 293 cells, the exogenous *GAA* hybrid transcript was under the control of SV40 promoter, a more standardized condition that reduces variability in expression of the minigene. As a result, this may favour a dose-dependent response to the *GAA*-tailored U1+1 snRNA. On the other hand, treating patient-derived fibroblasts with the U1+1 snRNA targeting the endogenous *GAA* transcript presumably may not be as standardized as using the minigene. Given the substantial difference between the biological context of these two experimental settings, it may therefore not be surprising that the dynamic of U1+1 effectiveness in promoting *GAA* exon 2 inclusion might differ. Nevertheless, the improvement of *GAA* exon 2 splicing process due to U1+1 snRNA treatment of c.-32-13T>G immortalized fibroblasts reflected in a concordant increase in GAA enzymatic activity, further confirming the accuracy of *GAA* exon 2 splicing in the physiologic context, as already observed in the minigene system. Moreover, this finding was nicely reproduced in primary c.-32-13T>G fibroblasts electroporated with the U1+1-expressing plasmid in which a 50% increase in GAA enzymatic activity was observed, even with the lowest U1+1 dose. We believe that, taken together, all these data suggest that the lower dose of U1+1 is already sufficient to reach the maximal effect of GAA exon 2 inclusion and GAA enzymatic activity in patient-derived fibroblasts. Considering that patients carrying the c.-32-13T>G variant often present a significant residual activity (up to 30%) (Niño et al. 2021), it is likely that the observed increase in the expression of functional *GAA* mRNA and GAA enzymatic activity following U1+1 snRNA treatment would be enough to exceed the threshold activity needed to prevent pathological glycogen accumulation and achieve a beneficial effect in clinical settings. Moreover, it is important to note that the increase of normally spliced *GAA* mRNA obtained with this *GAA* 5’ss specific U1 approach was similar to the increase observed using antisense oligonucleotides targeting exonic splicing silencers within *GAA* exon 2, that proved successful in diminishing the glycogen content in patient-derived myotubes carrying the c.-32-13T>G mutation (Goina et al. 2017).

Notably, we’ve found that U1+1 snRNA was able to partially recover the inclusion of *GAA* exon 2 also in the presence of mutations falling within or nearby the 3’ss, such as the c.-32-3C>G, supporting a role for engineered U1 snRNAs in promoting the selection from a distance of weakened 3’ss, as already suggested for *CFTR* (Donegà et al. 2020) or *SMN1* (Singh and Singh 2019) genes.

On the contrary, U1+1 favored the cryptic 3’ss use without improving the inclusion of the entire *GAA* exon 2 in the context of the c.503G>C missense variant and a still different scenario was observed when considering variants affecting the 5’ss such as c.546G>A, c.546G>C and c.546G>T synonymous variants for whom the treatment with U1+1 snRNA reproducibly improved their normal RNA processing while not empowering the cryptic 3’ss. As expected, a less evident effect was observed when U1+1 snRNA was employed to correct 5’ss variants that disrupt the highly conserved GU nucleotides at position +1 or +2.

It is interest to point out that Matos and colleagues have shown the feasibility of using modified U1 snRNAs to partially rescue normal splicing of transcripts bearing variants at the 5’ss. Furthermore, as these authors showed, the higher was the degree of homology between the mutated 5’ splice site and the specific U1 5’ tail, the higher was the ability to rescue the defective splicing (Matos et al. 2014). It is therefore possible to speculate that the little effect exerted by the U1+1 snRNA used in this work on intronic variants affecting the 5’ ss of exon 2 might be due to the degree of mismatches between the 5’ss and the modified U1. However, even under this condition some rescue of exon 2 inclusion was observed, suggesting that there might be room from improvement by optimizing the U1 design.

Altogether, these findings highlight that the effect of a specific modified U1 molecule is highly mutation-dependent and may rely also on the presence of other *cis*- or *trans*-acting splicing factors that, in concert, contribute to the exon selection and eventually orchestrate the splicing outcome. Nevertheless, AAV-mediated modified U1 delivery have been already tested *in-vivo* for the correction of splicing defects in several disease mouse models, demonstrating their effective potential coupled to a very low rate of off-targets and a good safety profile (Balestra et al. 2020; Donadon et al. 2019; Swirski et al. 2023).

## Conclusions

The data presented here suggest that the use of modified U1 molecules, aimed at strengthening the specific recognition of *GAA* exon 2 5’ss by the spliceosome machinery, represents a promising strategy for rescuing normal splicing of transcripts carrying both common and rare splicing variants affecting *GAA* exon 2 splicing. In particular, this approach might represent an interesting therapeutic option for patients carrying the common c.-32-13T>G variant, present in 90% of patients affected by the LO form of the disease, often displaying a relatively high residual activity, for which a therapeutic threshold can be achieved.

## Supplementary Information

Additional file 1.

Additional file 2.

## Data Availability

The authors confirm that the data supporting the findings of this study are available within the article. If additional data were required, it may be requested to the corresponding authors AD and EB.

## Abbreviations

*3’ss*: 3’ Splice site
*5’ss*: 5’ Splice site
*AMO*: Antisense morpholino oligonucleotide
*ERT*: Enzyme replacement therapy
*GAA*: Acid alpha-glucosidase
*IO*: Infantile onset
*LO*: Late onset
*LSD*: Lysosomal storage disease
*M6P*: Mannose-6-phosphate
*PD*: Pompe disease
*snRNA*: Small nuclear RNA
*snRNP*: Small nuclear ribonucleoprotein
*SV2*: Splice variant 2
*SV3*: Splice variant 3
