# Supplementary material for: Rescue of common and rare exon 2 skipping variants of the GAA gene using modified U1 snRNA
Source: Mol Med. 2025 Feb 4;31:45. doi: 10.1186/s10020-025-01090-z (PMC11796170; doi:10.1186/s10020-025-01090-z)
Supplement: Supplementary file 1 — Additional file 1. [file 10020_2025_1090_MOESM1_ESM.pdf]

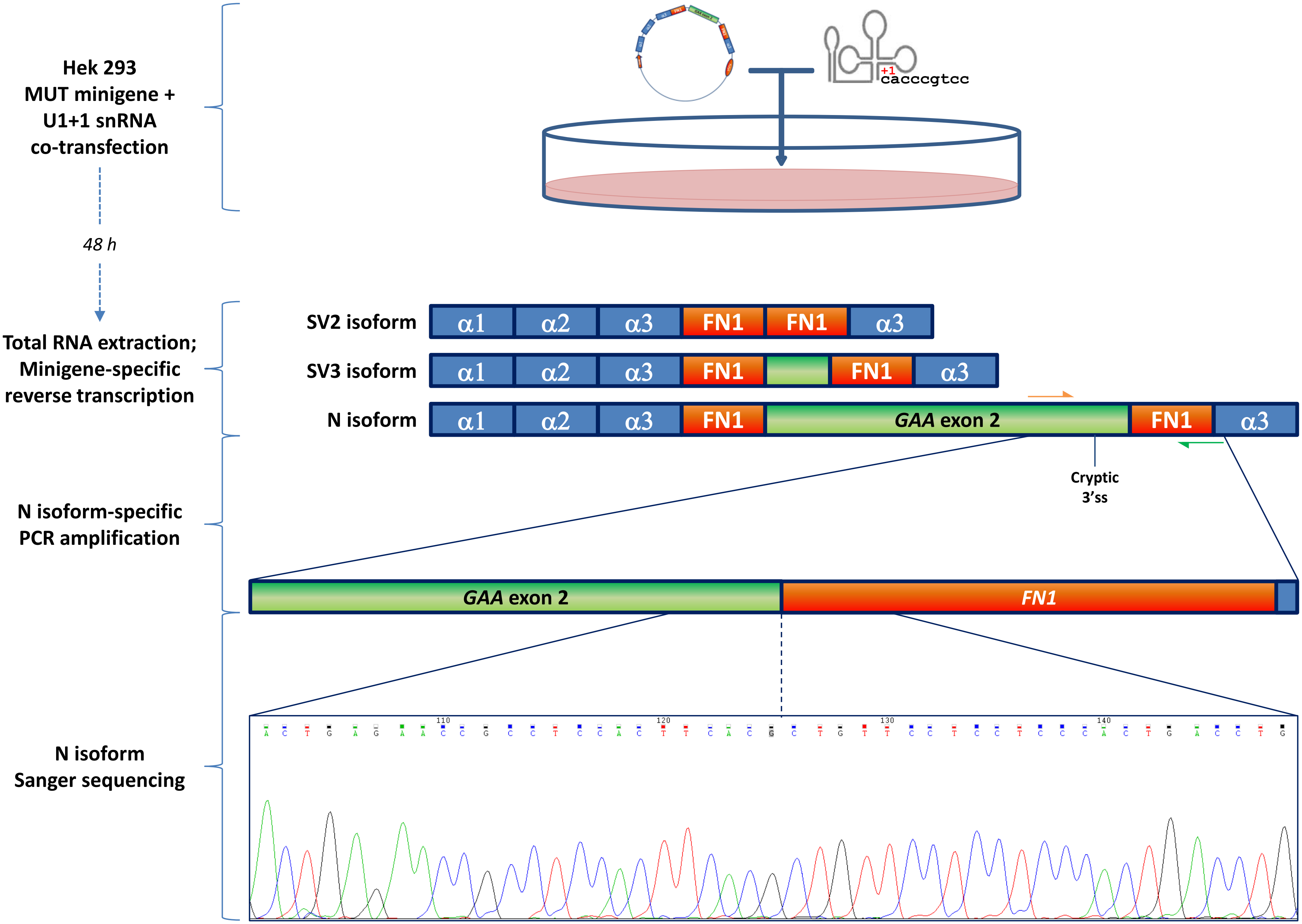
Hek 293
MUT minigene +
U1+1 snRNA
co-transfection
48 h
Total RNA extraction;
Minigene-specific
reverse transcription
N isoform-specific
PCR amplification
N isoform
Sanger sequencing
+1
caccсgtcc
SV2 isoform
α1 α2 α3 FN1 FN1 α3
SV3 isoform
α1 α2 α3 FN1 FN1 α3
N isoform
α1 α2 α3 FN1 *GAA* exon 2 FN1 α3
Cryptic
3’ss
GAA exon 2
FN1
110
120
130
140

Additional figure 1: Sanger sequencing of the exon 2-included *GAA* mRNA obtained from Hek 293 cells co-transfected with the c.-32-13T>G (MUT) minigene and U1+1 modified snRNA. Total RNA was reverse transcribed with Glo800Rbis minigene-specific primer and amplified using GoTaq MasterMix (Promega – Madison, Wisconsin) and GAA B forward primer (5'-CCACCCAGCTACCCCAGCTAC-3', matching 159 bp before the end of *GAA* exon 2, orange half-arrow) and BRA2 reverse primer (5'-TAGGATCCGGTCACCAGGAAGTTGGTTAAATCA-3', annealing to the fibronectin exon, green half-arrow). The following conditions were adopted for the amplification: initial denaturation = 95°C for 3 min; thermocycling = (95°C for 30 sec, 60°C for 30 sec, 72°C for 30 sec) for 35 cycles; final elongation = 72°C for 7 min. The PCR product was purified using ExoProStar™ 1-step (Cytiva – Marlborough, United States), sequenced using BigDye Terminator v3.1 (ThermoFisher Scientific – Waltham, Massachusetts) following manufacturer's instructions and resolved by capillary electrophoresis in an ABI 3500xL genetic analyzer (Applied Biosystems – Waltham, Massachusetts).
