# Supplementary material for: Rescue of common and rare exon 2 skipping variants of the GAA gene using modified U1 snRNA
Source: Mol Med. 2025 Feb 4;31:45. doi: 10.1186/s10020-025-01090-z (PMC11796170; doi:10.1186/s10020-025-01090-z)
Supplement: Supplementary file 2 — Additional file 2. [file 10020_2025_1090_MOESM2_ESM.pdf]

A

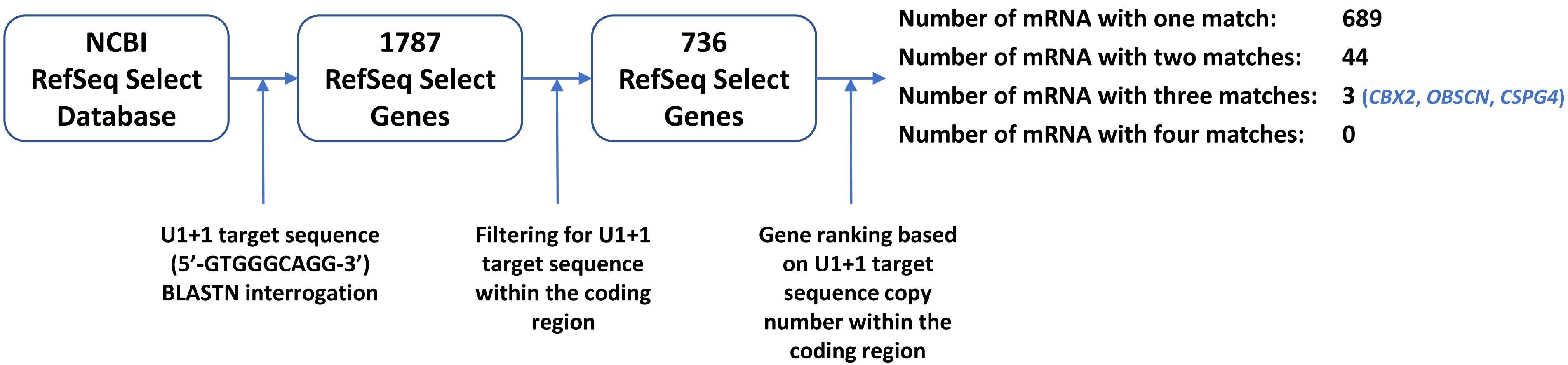
NCBI RefSeq Select Database
1787 RefSeq Select Genes
736 RefSeq Select Genes
U1+1 target sequence (5'-GTGGGCAGG-3') BLASTN interrogation
Filtering for U1+1 target sequence within the coding region
Gene ranking based on U1+1 target sequence copy number within the coding region
Number of mRNA with one match: 689
Number of mRNA with two matches: 44
Number of mRNA with three matches: 3 (CBX2, OBSCN, CSPG4)
Number of mRNA with four matches: 0

B

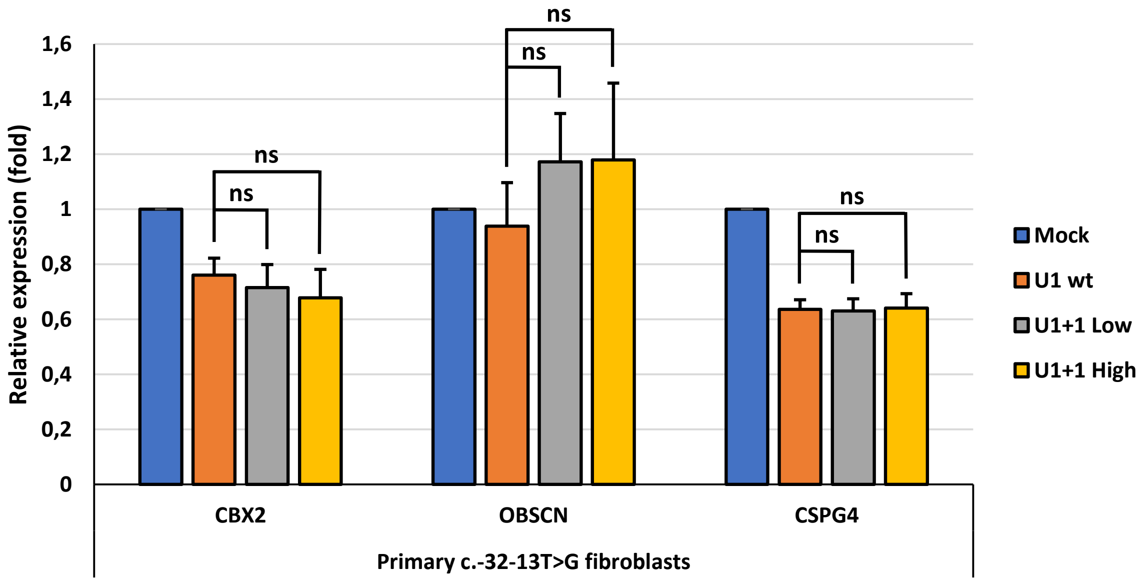
Relative expression (fold)
1,6
1,4
1,2
1
0,8
0,6
0,4
0,2
0
ns
CBX2
OBSCN
CSPG4
Primary c.-32-13T>G fibroblasts
Mock
U1 wt
U1+1 Low
U1+1 High

Additional figure 2: Bioinformatic identification and expression levels of the most likely U1+1 snRNA off-target genes in U1+1-electroporated c.-32-13T>G primary fibroblasts. (A) Bioinformatic workflow employed to identify the U1+1 snRNA most likely off-target transcripts. BLASTN (https://blast.ncbi.nlm.nih.gov/Blast.cgi?PROGRAM=blastn&BLAST_SPEC=GeoBlast&PAGE_TYPE=BlastSearch) was used to recover all the human transcripts from the RefSeq_Select database that match 100% with the 9 bp-long U1+1 target sequence (GTGGGCAGG) present at the 5' splice site of *GAA* exon 2. The retrieved transcripts were successively filtered for the presence of the target sequence within their coding region and then ranked on the basis of the copy number of the target sequence within the coding region of each transcript. **(B)** The relative expression levels of the three most likely U1+1 off-target genes in U1+1-electroporated c.-32-13T>G primay fibroblasts were assessed by real-time qPCR using Sso Advance Universal SYBR Green Supermix (Bio-Rad – Hercules, California) and the following primers: CBX2 FOR (5'-GGAACATGAGAAGGAGGTGC-3'), CBX2 REV (5'-GCTGGACTTGGATTTGGAGG-3'), OBSCN FOR (5'-TCTGGATGTCAAAGAGCCCA-3'), OBSCN REV (5'-TGTACCACATCACCTCCGTC-3'), CSPG4 FOR (5'-TGAGATCAGAAGGGACCAGC-3') and CSPG4 REV (5'-CCATCACCAGGTAGCCGG-3'). U1 wt electroporation was used as the negative snRNA control condition. Data were normalized for the expression of *HPRT* house-keeping gene and *CBX2*, *OBSCN* and *CSPG4* relative expression levels were calculated by applying the $\Delta\Delta$ct equation, considering the not electroporated primary fibroblasts as the reference sample. The results are expressed as mean ± SD of three independent experiments. Statistical analysis was conducted using Student t-test (ns = not significative).
